# Host blood-based biosignatures for subclinical TB and incipient TB: A prospective study of adult TB household contacts in Southern India

**DOI:** 10.3389/fimmu.2022.1051963

**Published:** 2023-01-11

**Authors:** Dhanasekaran Sivakumaran, Synne Jenum, Aashish Srivastava, Vidar M. Steen, Mario Vaz, Timothy Mark Doherty, Christian Ritz, Harleen M. S. Grewal

**Affiliations:** ^1^ Department of Clinical Science, Bergen Integrated Diagnostic Stewardship Cluster, Faculty of Medicine, University of Bergen, Bergen, Norway; ^2^ Department of Microbiology, Haukeland University Hospital, University of Bergen, Bergen, Norway; ^3^ Department of Infectious Diseases, Oslo University Hospital, Oslo, Norway; ^4^ Genome Core Facility, Clinical Laboratory (K2), Haukeland University Hospital, University of Bergen, Bergen, Norway; ^5^ Department of Physiology, St. John’s Medical College and Division of Health and Humanities, St. John’s Research Institute, Koramangala, Bangalore, India; ^6^ GlaxoSmithKline Vaccines, Wavre, Belgium; ^7^ National Institute of Public Health, University of Southern Denmark, Copenhagen, Denmark

**Keywords:** tuberculosis, incipient TB, subclinical TB, host-derived biological markers, RNA-seq - RNA sequencing

## Abstract

A large proportion of the global tuberculosis (TB) burden is asymptomatic and not detectable by symptom-based screening, driving the TB epidemic through continued *M. tuberculosis* transmission. Currently, no validated tools exist to diagnose incipient and subclinical TB. Nested within a large prospective study in household contacts of pulmonary TB cases in Southern India, we assessed 35 incipient TB and 12 subclinical TB cases, along with corresponding household active TB cases (n=11), and household controls (n=39) using high throughput methods for transcriptional and protein profiling. We split the data into training and test sets and applied a support vector machine classifier followed by a Lasso regression model to identify signatures. The Lasso regression model identified an 11-gene signature (*ABLIM2, C20orf197, CTC-543D15.3, CTD-2503O16.3, HLADRB3, METRNL, RAB11B-AS1, RP4-614C10.2, RNA5SP345, RSU1P1*, and *UACA*) that distinguished subclinical TB from incipient TB with a very good discriminatory power by AUCs in both training and test sets. Further, we identified an 8-protein signature comprising b-FGF, IFNγ, IL1RA, IL7, IL12p70, IL13, PDGF-BB, and VEGF that differentiated subclinical TB from incipient TB with good and moderate discriminatory power by AUCs in the training and test sets, respectively. The identified 11-gene signature discriminated well between the distinct stages of the TB disease spectrum, with very good discriminatory power, suggesting it could be useful for predicting TB progression in household contacts. However, the high discriminatory power could partly be due to over-fitting, and validation in other studies is warranted to confirm the potential of the immune biosignatures for identifying subclinical TB.

## Introduction

The covid-19 pandemic has reversed the slow decline in global tuberculosis (TB) mortality obtained by meticulous efforts since the World Health Organization (WHO) declared TB a public health emergency in 1994 (1). Early identification and treatment of TB cases to limit transmission and reduce morbidity and mortality has been the cornerstone in obtaining TB control, and earlier case identification before contagious disease develops, would undoubtedly speed up the process of getting the TB epidemic back on track after the devastating effect of the covid-19 pandemic (2, 3).

Following *M. tuberculosis* (*Mtb*) infection, a complex and dynamic host-pathogen interaction results in the TB disease spectrum ranging from asymptomatic latent infection to progressive TB disease. Despite extensive research, the complete picture of factors that favor mycobacterial control or host susceptibility for disease progression remains elusive (4). The now established broader understanding of a dynamic TB pathogenesis rather than a stringent view of latent or active TB challenges established treatment paradigms and calls for new diagnostic and therapeutic approaches (5). The progression from *Mtb* infection to active TB proceeds through intermediate asymptomatic stages, for which the notations incipient TB and subclinical TB have been suggested (6). Drain PK et al., have proposed a definition of four distinct stages of the TB disease spectrum: namely latent TB, incipient, subclinical TB and active TB. The suggested definitions are based on clinical, microbiological, immunological and radiological findings, but objective measurements of all these factors, for instance, bacterial metabolic activity partly overlap, and are indeed challenging (5).

Established tools that provide indirect evidence of *Mtb* infection are available [the tuberculin skin test (TST) and/or interferon-gamma release assays (IGRAs)] but have a poor predictive capacity for progression to active TB (7, 8) that do not seem to improve with more recent tests following the same principle (9–13). Active TB is clearly defined by symptomatic disease and identification of *Mtb* (confirmed TB) and/or the presence of typical radiological/histological findings. On the other hand, diagnostic tools are lacking for incipient and subclinical TB, and the scarcity of longitudinal clinical data does not allow a conclusion on whether these states represent a temporary lack of immunological control or a certain pre-state of active TB (14). The WHO and other stakeholders strongly encourage the development of novel tests capable of identifying incipient and subclinical TB (15, 16) in order to initiate tailored therapy to prevent TB progression and reduce *Mtb* transmission (17, 18). To meet the targeted product profile (TPPs), a rapid biomarker-based test should ideally be i) instrument-free or feasible with limited instrumentation and ii) based on easily accessible samples such as blood, urine, or breath (19, 20), and iii) discriminate between all the distinct stages of the TB disease spectrum. Multiple TB research groups are indeed making progress in identifying host biomarker-based risk signatures for TB progression (21, 22). However, the validation and approval according to the defined TPP will take time (15, 16). Blood transcriptomic studies have identified signatures of the risk of TB progression. A 16-gene signature of TB risk was identified by Zak et al. (18),, a 4-gene RISK4 signature by Suliman et al. (23), and a 6-gene RISK6 signature by Penn-Nicholson et al. (24),. RISK11 derived from Zak16 signature performed well as a non-sputum screening diagnostic test for active TB in case-control studies (25-27), and as a predictive test for TB progression within the following 6- and 12-months (27). Metabolic profiling also seems to have the potential to predict TB progression 1-year prior to TB diagnosis (28). A recent systematic review and meta-analysis evaluated 17 candidate mRNA signatures in a pooled dataset from four eligible studies comprising 1126 samples (29) and identified eight concise signatures that discriminated subjects with incipient TB from controls with very similar accuracy over a 2-year period. Unfortunately, none of the signatures met the TPP except when measured within three months of TB diagnosis.

To date, no clinical studies have assessed host-derived biomarkers across distinct stages of the TB disease spectrum, from *Mtb* infection, through incipient, subclinical, and active TB disease. Therefore, in a previously described prospective cohort (20) of TB index cases and their household contacts, we have conducted an in-depth analysis to elucidate the host transcriptional and protein characteristics across the TB disease spectrum, with an emphasis on biomarkers predicting TB progression in household contacts.

## Materials and methods

### Study design and participants

This case-control study was nested within a prospective observational cohort study of adult pulmonary TB (PTB) index cases and their household contacts conducted at our study site at the Emmaus Swiss Leprosy Project and Referral Hospital, in Palamaner and Kuppam Taluks, Chittoor district, Andhra Pradesh, India (3.200°N, 72.7500°E, altitude 683 m) between September 2010 and April 2012, to measure the incidence of *Mtb* infection and TB disease in a highly exposed population. Of 176 index cases aged >18 years and identified at the microscopy centers of the Revised National Tuberculosis Control Program (RNTCP; Government of India), 164 were recruited following written informed consent. In the context of our study, TB was confirmed in 150 cases by the presence of *Mtb* in sputum smear and/or culture-confirmed, as described previously (30). A total of 525 household contacts of the 176 index cases had no previous or current PTB, prophylactic or therapeutic TB treatment (exclusion criteria) and were recruited. The intended follow-up for all household contacts was one year.

### Clinical assessments and sampling

#### Baseline assessments

Medical history (including BCG vaccination status, history of TB exposure, prior TB/TB treatment (index cases only), and risk factors such as diabetes, smoking and alcohol consumption), sociodemographic, anthropometric, and clinical data were recorded. At baseline, a tuberculin skin test (TST) was performed by a trained nurse (2 TU/0.1 mL tuberculin; Span Diagnostics, Surat, India) and read after 48–72 hours; an induration ≥10 mm was defined as positive. Three independent radiologists interpreted the CXR (anterioposterior view), and agreement by at least two radiologists was required for the radiological diagnosis of PTB. Since the prevalence of HIV in India is low [<0.5% at the time (31)], HIV testing was not a criterion for inclusion. Nevertheless, following pre-test counseling, all study participants were offered HIV testing.

#### Longitudinal sampling

Sputum samples were collected from all household contacts at 0, 2, 6, and 12 months (two samples on two consecutive days at baseline, one sample at the remaining time points) were evaluated by smear microscopy for acid-fast bacilli (AFB) and cultured on both liquid (BACTEC MGIT 960™ [Becton and Dickinson, USA]) and solid (Lowenstein-Jensen) media as described previously (32). Positive cultures were confirmed using the HAIN kit (GenoType MTBC, Hain Lifescience GmbH, Nehren, Germany). CXR was repeated at the end of the study for all participants. Peripheral blood (~2.5 ml) was drawn for biomarker analysis at 0, 2, 6, and 12 months in the PAXgene Blood RNA tubes (PreAnalytiX, Hombrechtikon, Switzerland) and stored at -80°C until RNA extraction (PAXgene Blood RNA kit; PreAnalytiX, Hilden, Germany). Subsequently, 3 ml of blood was collected (1 ml each collected in Nil, TB antigen, and Mitogen tubes) for the QuantiFERON^®^ -TB Gold In-Tube (QFT-GIT) test (Cellestis, Australia) at 0, 2, 6, and 12 months.

### Assignment and definition of incipient and subclinical TB cases in the household contacts

**Figure 1:**
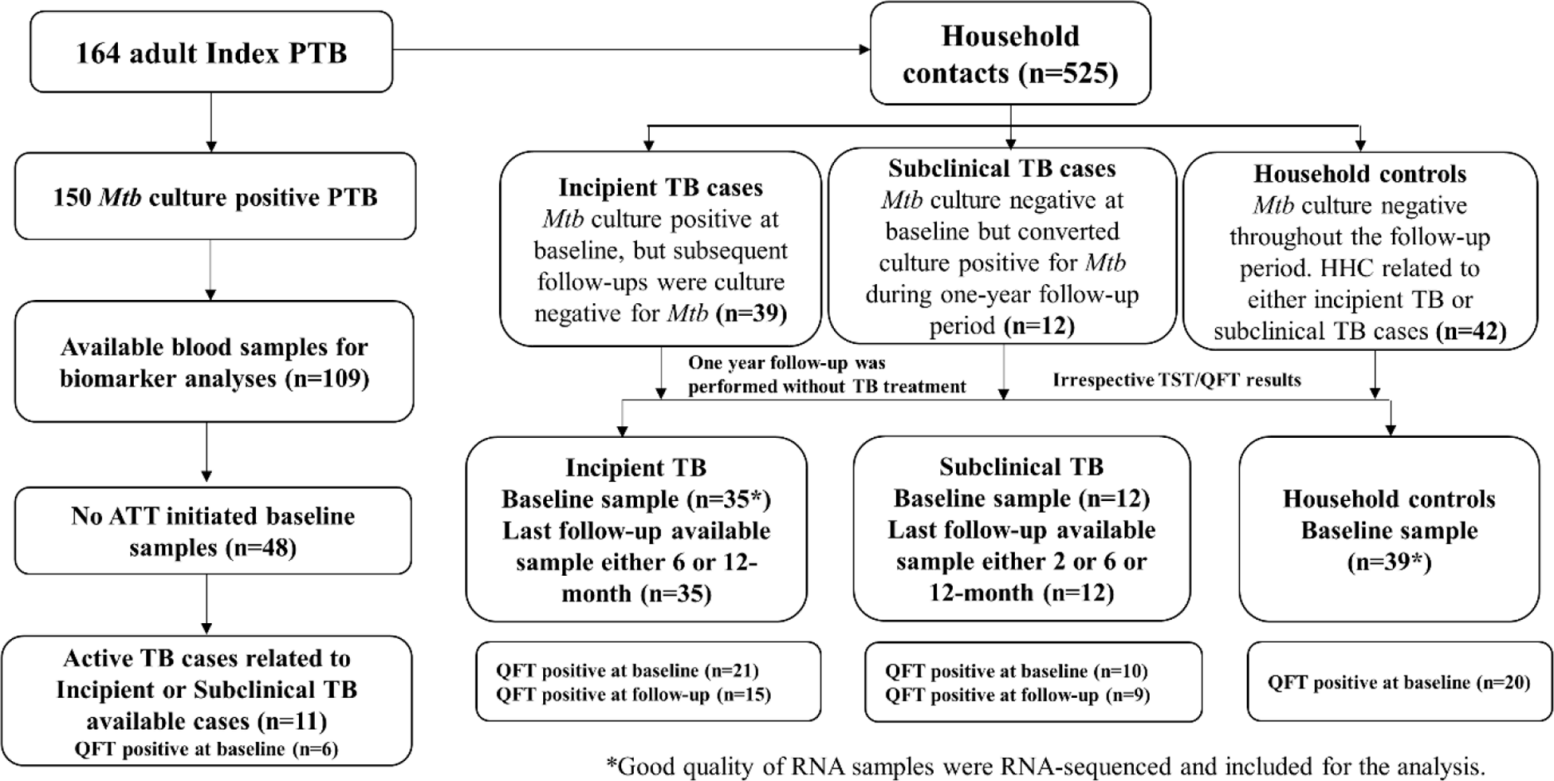
Study flow chart.

Although the concept of the TB disease spectrum and a dynamicity between disease states within the spectrum is an established theory (14, 15), verifiable criteria to distinguish between states do not exist. Notably, transient shedding of *Mtb* shortly after infection is likely in recently exposed household contacts and does not necessarily correlate to disease progression. Therefore, we took advantage of the longitudinal 1-year follow-up, assigning household contacts who progressed from negative to positive *Mtb* culture, indicating that immunological control was not obtained, to the category **Subclinical TB (n=12)**. The 12 *Mtb* culture-positive subclinical TB cases were referred to the RNTCP center for further assessment. Others have defined subclinical TB as “*subclinical TB disease due to viable Mtb bacteria that does not cause clinical TB-related symptoms but causes other abnormalities that can be detected using existing radiologic or microbiologic assays*” *(* 5). Samples for transcriptomic and protein signature identification representative of this state were selected from baseline and follow-up visits corresponding to the first positive *Mtb* culture. In contrast, household contacts that naturally controlled the infection during the 1-year follow-up, proven by 1) spontaneous conversion from positive *Mtb* cultures at baseline to negative cultures at later follow-up or 2) CXR abnormal for TB at baseline, but no progression to clinical disease and absence of positive *Mtb* cultures during 1-year follow-up were assigned to the category **Incipient TB (n=39;** only *Mtb* culture positive cases were referred to the RNTCP center for further assessment). Incipient TB has been defined by others as an “***asymptomatic*** *, early* ***pre-clinical disease*** *during which pathology evolves, such as mycobacterial replication or the inflammatory response. Radiological abnormalities or positive microbiological tests may or may not be present. This state may either evolve and lead to symptomatic clinical TB or regress and remain asymptomatic*” *(* 15). None of the *Mtb* culture-positive incipient TB cases received TB treatment during the one-year study period. Samples for transcriptomic and proteomic biomarkers analysis were selected from baseline and the last available follow-up visit (**Figure 1**).

### Selection of household controls

For the selection of household controls for the biomarker studies, we planned to include at least one age-matched household control from each family with a subclinical or an incipient TB case. However, this could not be achieved in all cases. Therefore, to maintain a 1:1 case-control ratio (n=42), we selected more than one household control from the same family of an incipient or subclinical TB case (from 12 families). Baseline samples were used for both transcriptomic and proteomic biomarker analysis.

### Selection of active TB cases among the adult index PTB

Active TB cases were selected using the same approach as household controls, with the intent of matching, where possible, each case with at least one age-matched household control from each family with a subclinical or an incipient TB case. In the study cohort, 33 out of 164 index TB cases were from families with either a subclinical and/or an incipient TB case. However, from these 33 index TB cases, blood samples from only 11 could be obtained before initiating TB treatment, and biomarker analysis was therefore limited to these 11 Active TB cases (**Figure 1**).

### Additional follow-up of household contacts with *Mtb* positive culture

Household contacts with *Mtb* positive culture at any time during the 1-year follow-up (subclinical and incipient TB) had an additional follow-up (not included in the original study protocol) at ~4-years of study completion. Our study team achieved this follow-up by directly visiting the participants’ homes, including 27 of 39 (69.2%) incipient and 11 of 12 (91.7%) subclinical TB cases.

### Total RNA extraction

Total RNA concentration and purity were measured using a Nanodrop spectrophotometer (Thermoscientific, Wilmington, DE, USA) and ranged between 0.4 –13.2µg (average 3.8 ± 1.65µg). In addition, RNA Integrity Number (RIN) was evaluated using an Agilent 2100 Bioanalyzer; In only seven samples, the RIN value showed ≤ 4.0; nevertheless, these seven samples were also analysed by RNA-sequencing.

### RNA sequencing and data management

Total RNA was extracted from PAXgene blood RNA tubes (n=151). Globin transcript depletion followed by cDNA library preparation using NuGen (now Tecan) Universal Plus mRNA-Seq with Human globin AnyDeplete. RNA-Sequencing was performed by Expression Analysis Inc., at ~60 million 2x75bp paired-end reads on Illumina HiSeq-4000 sequencers (n=151). Raw RNA-Seq reads were aligned to human genome GRCh38.p13 using hisat 2.0.5 with Gencode v26 gene transfer format (GTF) file for reference annotation (33), and were quantified using Feature Counts. Subsequently, read counts were normalized and log 2 transformed using the DESeq2 R package. After the initial assessment, we noticed that seven samples with low RIN values (3-control baseline samples; 2-incipient TB baseline sample; 1-incipient baseline and follow-up samples) yielded poor quality of sequencing data. These seven samples were therefore excluded from further analysis. A total of 35 incipient TB cases, 12 subclinical TB, 11 active TB, and 39 household controls were included for the transcriptional and proteomic analysis (**Figure 1**).

### Multiplex cytokine/chemokine assays

Biomarkers at the proteomic level were analysed in unstimulated supernatants (nil tubes) from the QFT-GIT assay [n=147] by a 27-plex Interleukin (IL)-1β, IL-1 receptor antagonist (IL1-RA), IL-2, IL-4, IL-5, IL-6, IL-7, IL-8/CXCL8, IL-9, IL-10, IL-12 (p70), IL-13, IL-15, IL-17, CXCL11, bFGF, G-CSF, GM-CSF, IP-10, MCP-1/CCL2, MIP-1α/CCL3, MIP-1β/CCL4, PDGF-BB, RANTES/CCL5, TNF, and VEGF cytokine/chemokine kit (Bio-Rad Laboratories Inc., California, USA) according to the manufacturer’s instructions. Data acquisition was performed on a Luminex100 analyser (Luminex Corporation, Austin, Texas, USA) according to the manufacturer’s instructions. Cytokine/Chemokine concentrations were measured in pg ml^-1^.

### Statistical methods

Differences in baseline characteristics were identified by applying Pearson’s chi-square or Fisher’s exact test and the Kruskal-Wallis test (for continuous variables) where appropriate. SPSS software version 28.0 was used.

The normalized and log2 transformed matrix data with all study samples was exported and analyzed by comparing the categorized TB disease states (Active TB, Subclinical TB, Incipient TB, Household controls) by applying the appropriate methods: The one-way analysis of variance (ANOVA) was used for comparisons across all study groups using Qlucore Omics Explorer V3.6 (Qlucore AB, Lund, Sweden). Subsequently, a comparison between subclinical and incipient TB was undertaken by univariate feature selection with a prebuilt t-test with correction for multiple testing applying the Benjamini-Hochberg method. The significance level was set to a p-value (≤0.01).

After identifying genes that were differentially expressed between TB disease states, the data set was split into a training set (2/3) and a test set (1/3), and the signature was identified by applying a support vector machine (SVM) classifier leave-one-out cross-validation. The prediction model was generated by a prebuilt linear SVM model in Qlucore Omics Explorer V3.6. Subsequently, the Lasso (Least absolute shrinkage and selection operator) regression analysis was applied to remove biomarkers with overlapping predictive ability. Finally, the diagnostic capacity of the identified signatures to correctly assign the participants as subclinical TB or incipient TB was evaluated by sensitivity, specificity and summarized by employing receiver operator characteristic (ROC) curves and area under the curve (AUC) values in the training set and subsequently in the test set using R (R Core Team) (34) through the graphical user interface RStudio (www.rstudio.com).

WebGestalt (WEB-based GEne SeT AnaLysis Toolkit) (35) is a widely used gene set enrichment tool that helps users extract biological insights from the selected genes of interest. The Reactome and Kyoto Encyclopedia of Genes and Genomes (KEGG) pathway databases were selected to show the pathway enrichment map of the selected genes. Reactome (36) is the most actively updated general-purpose public database of human pathways, while KEGG (37) is useful as it contains multiple pathways and includes disease-associated gene sets. The top results were ranked using the Benjamini–Hochberg method for controlling the false discovery rate. A p-value < 0.05 was considered significant.

### Ethics approval and consent to participate

Ethical approval for this study was obtained from the Institutional Ethical Review Board (IERB) of St. John’s Medical College, Bangalore (IERB/1/527/08 date 15.07.2008). The material transfer agreement between St. John’s Medical College, Bangalore and the University of Bergen, Norway, was obtained from the Department of Biotechnology, Government of India (No. BT/Med.II/Adv (SS)/Misc./02/2012). In addition, ethical approval was obtained from the Regional Committee for Medical and Health Research Ethics, Western-Norway (REK Vest; Ref no: **2018/1614 D**). Adults provided written informed consent. For children ≤7 years, parents/guardians provided written informed consent, and for participants >7 years, an additional written assent was obtained.

## Results

### Characterization of the household contacts

The detailed results of this cohort study characterization are described elsewhere (32). Both children and adults were included in the household contacts, whereas index TB cases were only adults. Thus, as expected, age and gender were significantly associated with active TB compared to the other distinct TB spectrum groups (**Table 1**); the subsequent analysis showed no association of age (p=0.32) and gender (p=0.89) between incipient and subclinical TB. The 12 subclinical TB cases had a mean age of 25.2 years (range 6-55), males constituted 33.3%, and 83.3% had a positive QFT-GIT at baseline. One subject had symptoms suggestive of TB at baseline (cough) that persisted at the 2-month follow-up visit. At the 2-month visit, 2 of the 12 cases had a CXR abnormal for TB (including the subject with persistent cough), but *Mtb* cultures did not turn positive until the 6-month visit. After one year, at the end-of-study, the remaining 10 cases were still asymptomatic despite the fact that two had positive *Mtb* cultures already at 6 months, and 8 subjects had positive *Mtb* cultures at 12 months. Two of the 10 also had CXRs abnormal for TB. All the 12 subclinical TB cases were referred to the RNTCP center at the time when a positive *Mtb* culture was known for evaluation for TB treatment initiation.

**Table 1:** Baseline characteristics of the study groups.

Clinical Characteristics	Active TB (n=11)	Subclinical TB at baseline (n=12)	Subclinical TB at follow-up (n=12)	Incipient TB at baseline (n=35)	Incipient TB at follow-up (n=35)	Household controls (n=39)	p-value
Demographics							
Age in years (mean)	42.3	25.2	–	26.0	–	26.3	0.027
Range	27 - 68	6 - 55	–	2 - 63	–	5 - 57	
Gender (Male, %)	9 (81.8)	4 (33.3)	–	19 (54.3)	–	10 (25.6)	0.003
Mycobacterial exposure							
Known BCG vaccination (%)	4 (36.4)	10 (83.3)	–	25 (71.4)	–	24 (61.5)	0.522
Unknown (%)	4 (36.4)	2 (16.7)	–	3 (8.6)	–	4 (10.2)	0.104
Tuberculin skin test							
Positive (≥10 mm) (%)	6 (54.5)	7 (58.3)	–	11 (31.4)	–	18 (46.2)	0.090
Median (mm)	15	15	–	13	–	13	
Quantiferon Gold in tube							
Positive (≥0.35 IU/mL) (%)	6 (54.5)	10 (83.3)	9 (75.0)	21 (60.0)	15 (42.8)	20 (51.3)	0.060^⫧^
Indeterminate/Not available (%)	1 (9.0)	1 (8.3)	2 (16.6)	2 (5.7)	2 (5.7)	0 (0.0)	0.769^⫧^
IFNg Median (IU/mL)	6.3	6.8	2.2	2.9	4.7	9.5	0.352^⫧^
Symptoms							
Cough ≥2 weeks (%)	3 (27.3)	1^$^(8.3)	1 (8.3)	0 (0.0)	1 (2.9)	0 (0.0)	<0.001
Fever ≥1 week (%)	9 (81.8)	0 (0.0)	0 (0.0)	0 (0.0)	0 (0.0)	0 (0.0)	<0.001
Weight loss (%)	7 (63.6)	1^$^ (8.3)	0 (0.0)	0 (0.0)	0 (0.0)	0 (0.0)	<0.001
Mtb culture							
Positive (%)	11 (100.0)	0 (0.0)	12^¤^ (100.0)	28 (80.0)	0 (0.0)	0 (0.0)	<0.001
Findings							
Abnormal Chest X-ray for TB (%)	11 (100.0)	2 (16.6)	4^*^(33.3)	7 (20.0)	0 (0.0)	0 (0.0)	<0.001
BMI Underweight^#^ (%)	8 (72.7)	9 (75.0)	–	18 (51.4)	11 (31.4)	21 (53.8)	0.180^⫧^

^#^For adults over 18 years of age <18 kg/m^2^, and for children 2-18 years of age <5% percentile was applied.

^*^For two cases, the chest X-ray was abnormal for TB at 2 months and whose blood samples collected at 2 months were used for biomarker analysis; for the remaining two cases, CXR was abnormal at 12-months.

^$^ Same subject had more than one symptom.

^¤^Culture positive for Mtb either at 6-month or 12-months.

^⫧^Baseline comparison.

Of 39 Incipient TB cases, 35 had available samples for biomarker analyses. The mean age was 26.0 years (range 2-63), males constituted 54.3%, and 60.0% had a positive QFT-GIT at baseline (**Table 1**). None of the 35 incipient TB cases had symptoms of TB at baseline. Except for one (who had a cough), the remaining 34 incipient TB cases did not develop TB-related symptoms during the 1-year follow-up. At baseline, 28 were categorized as incipient TB cases due to *Mtb* culture positivity, whereas seven were categorized due to CXR abnormal for TB (**Table 1**). Nine out of thirty-five incipient TB cases were QFT negative at baseline and consistently negative throughout the follow-up, except one, whose QFT result was unavailable for the follow-up period; three had QFT conversion to positive at 2-, 6- and 12 months. Notably, three had QFT reversion to negative during follow-up.

Household controls had a mean age of 26.3 (range 5-57), males accounted for 25.6%, and 51.3% had a positive QFT-GIT at baseline. Complete clinical characteristics are shown in **Table 1**.

According to our study protocol, all *Mtb* culture-positive participants were referred to the RNTCP center responsible for the decision-to-treat and further management. From thereafter, follow-up in the context of our study was stopped. Regrettably, there was no consistent back-reporting from the RNTCP to our study team. Approximately four years after study completion, our study team obtained follow-up information by a direct home visit to 38 of 41 *Mtb* culture-positive participants (11 subclinical TB, 27 incipient TB). After per-protocol study closure, 12 cases in whom TB treatment was not initiated when first referred to the RNTCP had progressed to active TB and received TB treatment (6 cases classified as subclinical TB and 6 cases classified with incipient TB). However, it was not possible to obtain information on time for diagnosis or treatment duration. Three subclinical TB cases had died from an unknown cause.

### Characterization of the Index TB patients

The 11 Index TB cases within households of subclinical and incipient TB cases had a mean age of 42.3 years (range 27-68), males accounted for 81.8%, and 54.5% had a positive QFT-GIT at baseline (**Table 1**).

### Transcriptional biosignatures

#### Identification of differentially expressed genes across distinct stages of the TB disease spectrum

Recent advancement in next-generation sequencing (NGS) has resulted in the ability to identify novel transcripts directly from sequencing data. For the 144 samples, our RNA-seq data can map 50898 Ensembl gene IDs containing 47443 unique genes and 3455 splice variants. The first method involved filtering the genes using Qlucore Omics explorer by applying ANOVA with a q-value cut-off of 0.05, which resulted in 10101 Ensembl gene IDs (9576 unique genes) that were differentially expressed between distinct stages across the TB disease spectrum (**Supplementary Table 1**).

#### Identification of a transcriptional signature discriminating subclinical TB from incipient TB at baseline contact investigation

**Figure 2:**
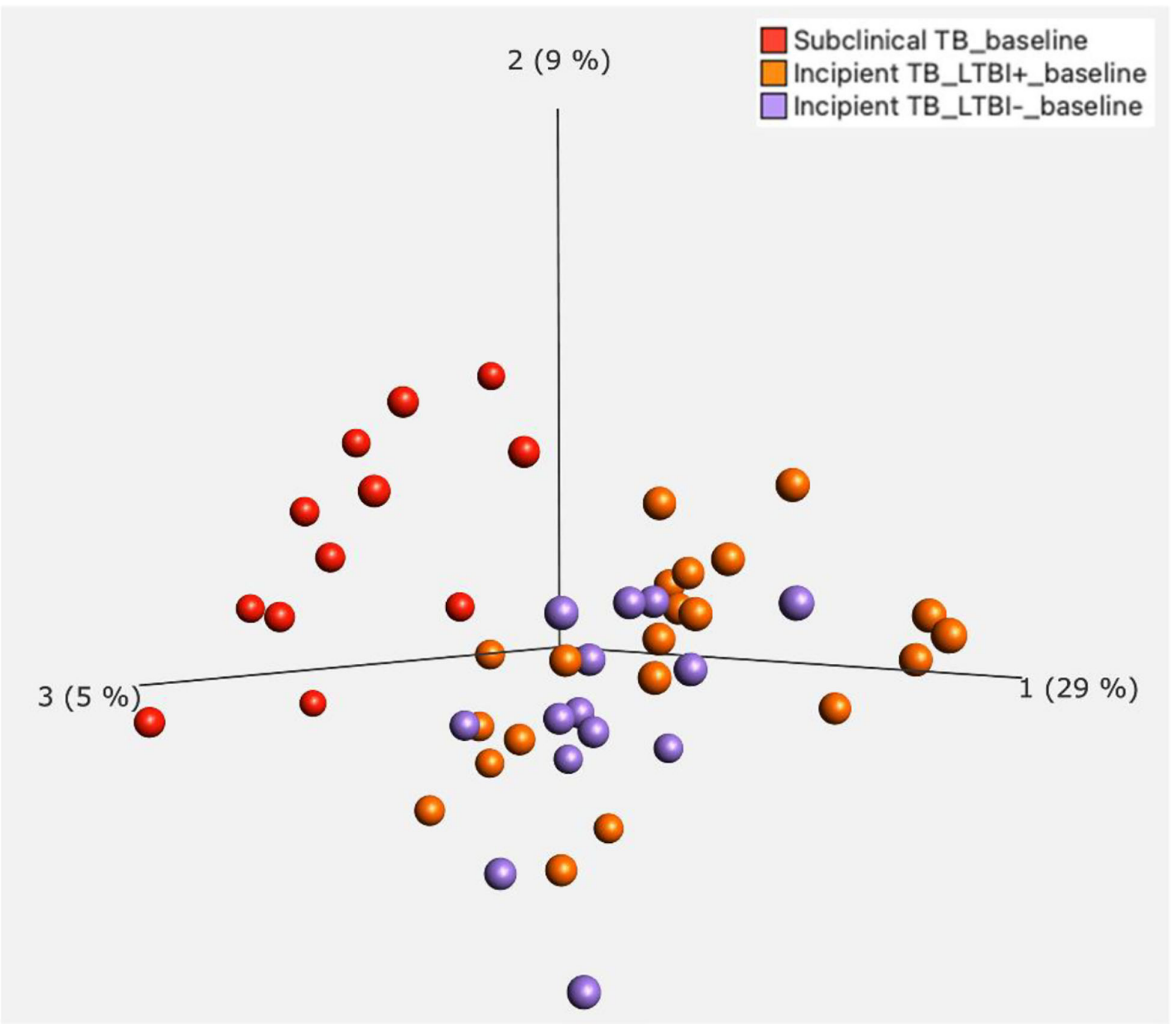
Principal Component Analysis (PCA) for incipient TB and subclinical TB.

**Figure 3:**
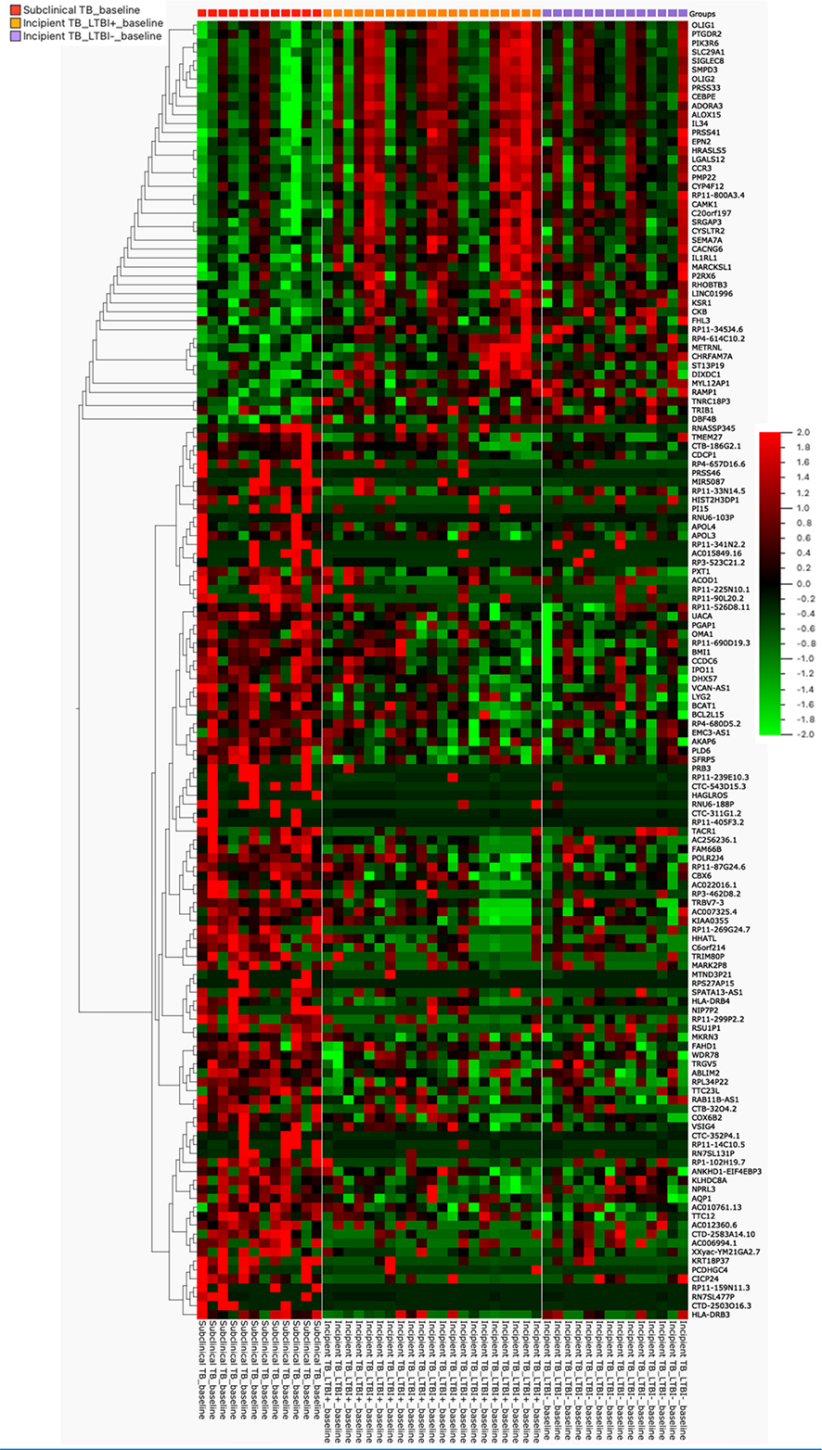
The heat map was generated for 145 genes, the columns represent genes, and the rows represent sample groups. Each cell is colorized based on the level of expression of that gene in that sample.

In our further approach, we focused on discriminating subclinical from incipient TB cases because of the obvious implications for follow-up and treatment, and their lack of clear “diagnostic criteria/tools”. We applied a univariate feature selection approach using a two-group comparison (t-test) with a stringent p-value (p ≤ 0.01). This analysis identified 145 unique genes (**Supplementary Table 2**) discriminating subclinical from incipient TB cases (**Figure 2**). A heat map and hierarchical clustering of the differentially expressed genes are displayed in **Figure 3**.

**Figure 4:**
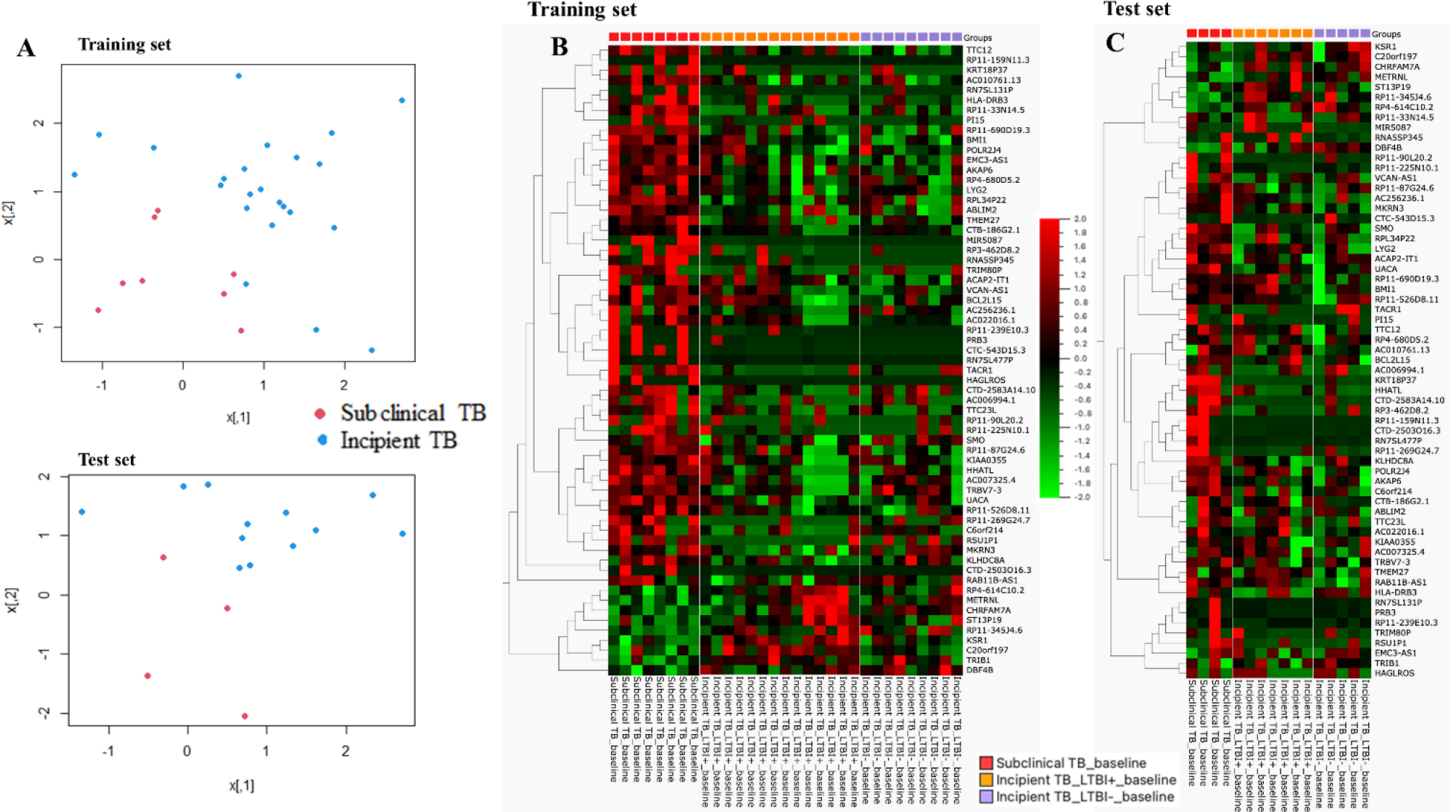
**(A)** Scatter plot of the two groups for training and test sets, respectively. **(B)** Training set: the heat map was generated for the 63-gene signature, the columns of the heat map represent genes, and the rows represent sample groups. Each cell is colorized based on the level of expression of that gene in that sample. **(C)** Test set: the heat map was generated for the 63-gene signature, the columns of the heat map represent genes, and the rows represent sample groups. Each cell is colorized based on the level of expression of that gene in that sample.

**Figure 5:**
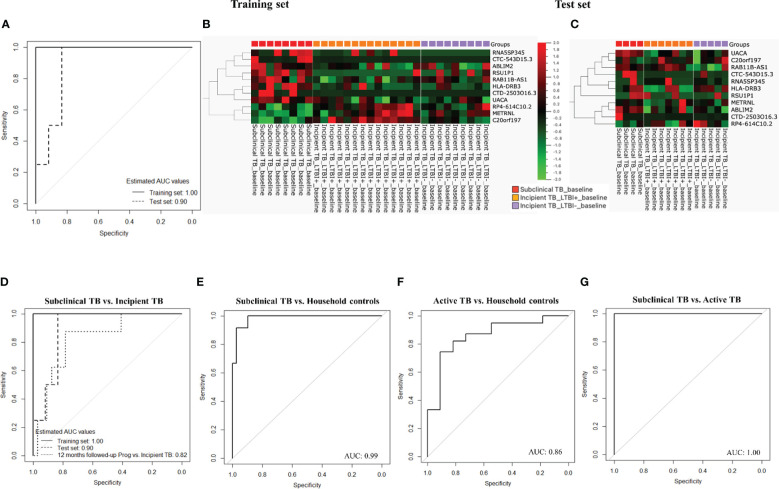
**(A)** ROC curves for gene signature discriminate subclinical TB from incipient TB. **(B)** Training set: Cluster heat map for the 11-gene signature, the columns of the heat map represent genes, and the rows represent sample groups. **(C)** Test set: Cluster heat map for the 11-gene signature, the columns of the heat map represent genes, and the rows represent sample groups. Each cell is colorized based on the level of expression of that gene in that sample. **(D)** ROC curves for the 11-gene signature distinguished subclinical TB from incipient TB. **(E)** ROC curves for the 11-gene signature distinguished subclinical TB from household controls. **(F)** ROC curves for gene signature that distinguishes active TB from household controls. **(G)** ROC curves for gene signature that distinguishes subclinical TB from active TB.

The dataset of subclinical and incipient TB was randomly split into a training (2/3) and a test set (1/3), and applying a support vector machine classifier, 63 genes were identified in the training set and later tested in the test set (**Figures 4A-C**; **Supplementary Table 3**). Finally, by Lasso regression, an 11-gene signature was identified in the training set, comprising *ABLIM2, C20orf197, CTC-543D15.3, CTD-2503O16.3, HLADRB3, METRNL, RAB11B-AS1, RP4-614C10.2, RNA5SP345, RSU1P1*, and *UACA* (**Table 2**; sensitivity 100.0% (95%CI, 63.1-100.0), specificity 100.0% (95%CI, 85.2-100.0, AUC of 1.00 (95% CI: 0.99 – 1.00)). The performance of the 11-gene signature in the test set was promising, with a sensitivity of 100.0% (95%CI, 39.8-100.0) and a specificity of 83.3% (95%CI, 51.6-97.9) corresponding to an AUC of 0.90 (95% CI: 0.74 – 1.00) (**Figures 5A-C**). In addition, based on the 4-year followed-up data, the 63-gene signature identified by SVM at baseline was assessed in the subclinical TB vs. incipient TB. The scatter plot represented the two groups at baseline and follow-up (**Supplementary Figure 1**).

**Table 2:** Expression and regression coefficients for each biomarker of the identified 11-gene signature by lasso regression.

Gene Expression	Genes	Slope co-efficient
Decreased	*C20orf197*	-1.43861
	*METRNL*	-0.70839
	*RP4.614C10.2*	-0.75841
Increased	*ABLIM2*	4.51912
	*CTC.543D15.3*	1.77813
	*CTD.2503O16.3*	1.45265
	*HLA.DRB3*	0.54132
	*RAB11B.AS1*	2.44220
	*RNA5SP345*	5.44111
	*RSU1P1*	1.44765
	*UACA *	2.51849

Next, the performance of the identified 11-gene signature was assessed in 6- and 12-month follow-up samples from subclinical and incipient TB cases, correctly classifying 4 of 12 subclinical TB and 29 of 32 incipient TB (sensitivity 33.3%, 95%CI, 10.0-65.1, specificity 90.6%, 95%CI, 75.0-98.0) providing an AUC of 0.75 (95% CI: 0.60 – 0.90). Subsequently, the performance of the 11-gene signature was assessed in the 12-month follow-up samples showed a slightly higher sensitivity of 50.0% (95%CI, 15.7-84.3), with the same specificity of 90.6%, 95%CI, 75.0-98.0) providing an AUC of 0.82 (95% CI: 0.67 – 0.98; **Figure 5D**).

#### Performance of the 11-gene signature across distinct stages of the TB disease spectrum at baseline

We then tested if the 11-gene signature identified based on differences between subclinical and incipient TB also discriminated subclinical TB from household controls. Gladly, the 11-gene signature correctly classified 12 of 12 subclinical TB cases and 33 of 39 household controls (sensitivity 100.0%, 95%CI, 73.5-100.0, specificity 84.6%, 95%CI, 69.5-91.4) corresponding to an AUC of 0.99 (95% CI: 0.96 – 1.00; **Figure 5E**). Subsequently, we assessed the 11-gene signature’s discriminatory capacity between active TB and household controls, obtaining correct classification in 11 of 11 active TB cases and 27 of 39 household controls (sensitivity 100.0%, 95%CI, 71.5-100.0, specificity 69.2%, 95%CI, 52.4-83.0) corresponding to an AUC of 0.86 (95% CI: 0.73 – 0.99; **Figure 5F**)

Next, we tested the capacity of the 11-gene signature to discriminate subclinical from active TB. We found correct classification of 12 of 12 subclinical TB and 11 of 11 active TB cases from (sensitivity and specificity of 100.0%, 95%CI, 71.5-100.0) corresponding to an AUC of 1.00 (95% CI: 0.99 – 1.00; **Figure 5G**).

**Figure 6:**
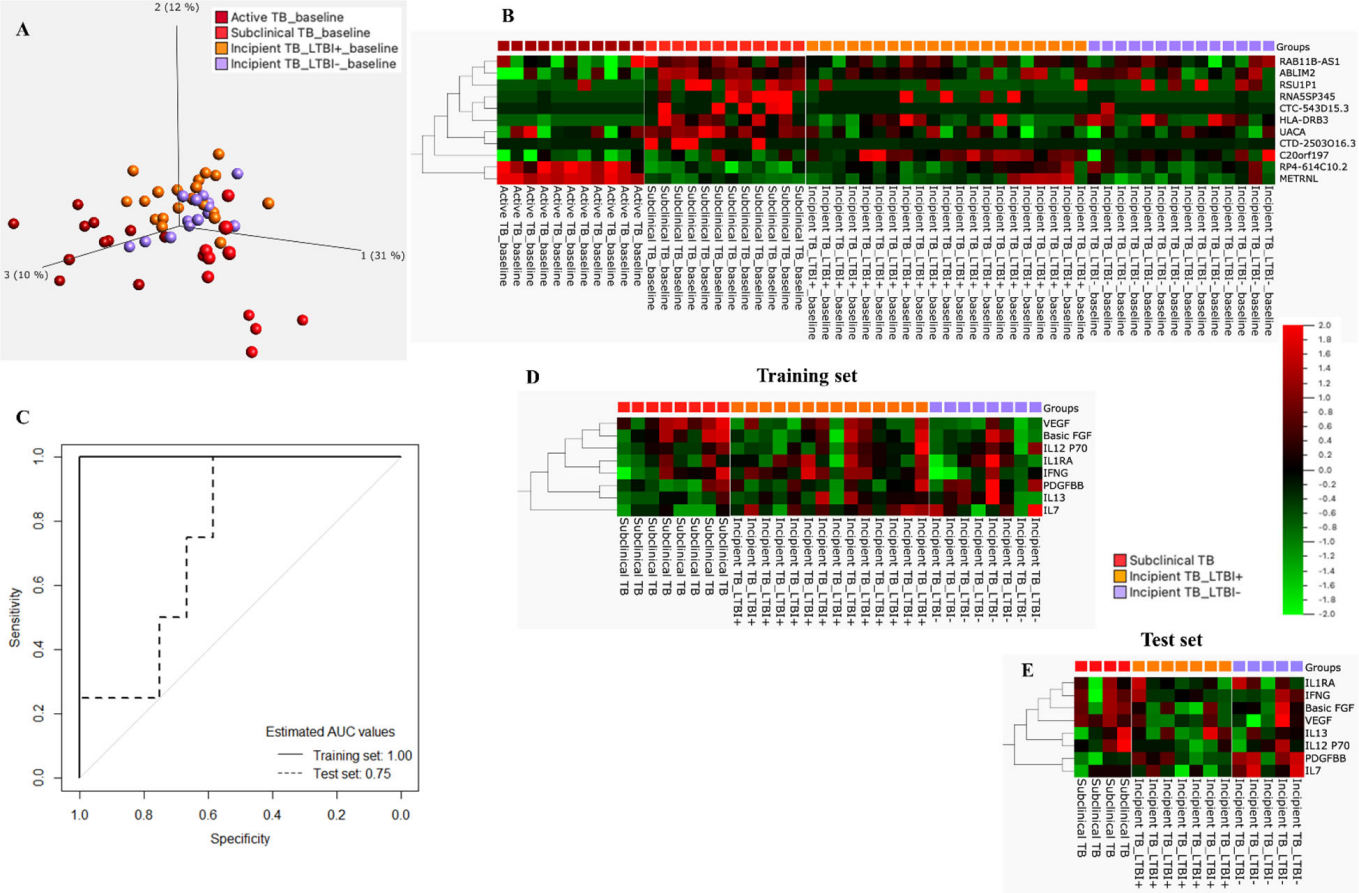
**(A)** PCA plot for incipient, subclinical, and Active TB. **(B)** Cluster heat map for the 11-gene signature, the columns of the heat map represent genes, and the rows represent sample groups. **(C)** ROC curves for the 8-protein signature distinguished subclinical TB from incipient TB. **(D)** Training set: Cluster heat map for 8-protein signature, the columns of the heat map represent cytokines/chemokines, and the rows represent sample groups. Each cell is colorized based on the protein concentration level of that sample. **(E)** Test set: Cluster heat map for the 8-protein signature, the columns of the heat map represent cytokines/chemokines, and the rows represent sample groups.

Last, the 11 genes constituting the signature were entered into a principal component analysis (PCA), confirming a distinct separation of all stages of the TB disease spectrum, namely Incipient TB, subclinical TB, and active TB (**Figure 6A**). Similar clustering was identified by applying a heatmap to the identified 11-gene signature (**Figure 6B**).

### Protein biosignatures

#### Identification of protein signature discriminating subclinical TB from incipient TB at baseline

The mean concentration (pg/mL) of the 27 protein biomarkers was measured in **Supplementary Table 4**. Following a similar approach for proteins, the start for biosignature discovery was discriminatory capacity between subclinical and incipient cases. Lasso regression analysis was applied directly to the 27 protein biomarkers identifying an 8-protein signature comprising b-FGF, IFNγ, IL1RA, IL7, IL12p70, IL13, PDGF-BB, and VEGF (**Table 3**) that discriminated subclinical TB from incipient TB in the training set (sensitivity 100.0%, 95%CI, 63.1-100.0, specificity 90.9%, 95%CI, 84.6-100.0; AUC of 1.00, 95%CI, 0.99-1.00). In the test set, the identified 8-protein signature correctly classifying 4 of 4 subclinical TB and 6 of 12 incipient TB (sensitivity 100.0%, 95%CI, 39.8-100.0, specificity 50.0%, 95%CI, 21.1-78.9) obtaining an AUC of 0.75 (95%CI, 0.50-0.99; **Figures 6C-E**).

**Table 3:** Expression and regression coefficients for each biomarker of the identified 8-protein signature by lasso regression.

Protein Expression	Proteins	Slope co-efficient*
Decreased	IFNG	-1.15457
	IL1RA	-0.29658
	IL7	-14.2179
	IL12p70	-1.89595
	IL13	-58.292
	PDGFBB	-0.05101
Increased	bFGF	40.0787
	VEGF	0.95258

*Slope coefficients are scaled-up by a factor of 1000.

Subsequently, the identified 8-protein signature was applied in samples from subclinical TB and incipient TB cases, obtained at the 1-year follow-up, correctly classifying 6 of 8 subclinical and 19 of 34 incipient TB cases (sensitivity 75.0%, 95%CI, 34.9-96.8, specificity 55.9%, 95%CI, 37.0-72.8) corresponding to an AUC of 0.64 (95% CI: 0.41 – 0.86; **Supplementary Figure 2A**).

#### Performance of the 8-protein signature across distinct stages of the TB disease spectrum at baseline

Then, the 8-protein signature was tested in subclinical TB vs. household controls, correctly classifying 12 of 12 subclinical TB, and 19 of 36 household controls (sensitivity 100.0%, 95%CI, 73.5-100.0, specificity 52.8%, 95%CI, 35.5-69.6) corresponding to an AUC of 0.71 (95% CI: 0.56 – 0.86; **Supplementary Figure 2B**). Similarly, the 8-protein signature was tested in active TB vs. household controls, correctly classifying 8 of 11 subclinical TB, and 19 of 36 household controls (sensitivity 72.7%, 95%CI, 39.1-93.8, specificity 52.8%, 95%CI, 35.5-69.6) corresponding to an AUC of 0.70 (95% CI: 0.54 – 0.86; **Supplementary Figure 2C**)

Further, the 8-protein signature was tested in subclinical vs. active TB, correctly classifying 8 of 11 active TB, and 12 of 12 subclinical TB (sensitivity of 72.7%, 95%CI, 39.0-94.0, specificity of 100.0%, 95%CI, 73.5–100.0) corresponding to an AUC of 0.93 (95% CI: 0.83 – 1.00; **Supplementary Figure 2D**)

#### Gene ontology term enrichment analysis

The 145 identified by the univariate feature selection approach were uploaded to the WebGestalt for Gene Ontology (GO), Reactome, and KEGG pathway enrichment analyses. The GO analysis revealed the key gene set to be enriched predominantly by genes involved in immune responses, more specifically, cytokine signaling, adaptive immune responses, signaling by interleukins, and G-alpha signaling events (**Supplementary Figure 3A**). Intriguingly, KEGG analysis results showed that the selected vital genes were also associated with TB (**Supplementary Figure 3B**).

## Discussion

Accurate recognition of these early disease states within the TB disease spectrum by novel diagnostic tools has the potential to intervene prior to progression to overt infectious TB reducing disease burden and transmission. The nature of the host response is reflected in gene expression. Over the past decade, RNA sequencing and microarray-based analysis have been applied in biomarker discovery and succeeded in identifying host mRNA signatures in whole blood with reasonable discrimination accuracy between subjects with active TB, *Mtb* infection, and other diseases (38). However, not all forms of TB share common disease pathogenesis (39), evidenced by the wide time interval between the first *Mtb* infection and active TB that can span from weeks to a lifetime (5). As a result, current approaches to risk stratification, including combining epidemiological parameters with or without IGRA, could potentially be improved. Nevertheless, in most TB-exposed subjects, screening for incipient and subclinical TB by host RNA signatures is likely relevant (27, 39, 40).

In the prospective cohort study of 525 household contacts, 7.5% [39] were categorized with incipient TB and 2.3% [12] with subclinical TB. Unlike previous studies, we have included and categorized several distinct stages across the TB disease spectrum as a basis for the NGS and study aims. We acknowledge the lack of a consensus definition and validated tools for this categorizing (5, 14), but the proportion of household contacts that progressed to TB (2.3%) is consistent with reports from the GC6-74 cohort (28). None of the 12 subclinical TB cases died in the one-year study period. However, our four-year follow-up revealed that 3 out of 12 subclinical TB cases died from unknown causes, indicating that very close follow-up from the healthcare system would benefit the number of patients needing to be treated to prevent TB. According to our categorization of incipient TB cases, these subjects had the transient presence of *Mtb* in the respiratory specimen at baseline contact investigation but later achieved immunological control. Only 60% had a positive QFT-GIT at baseline contact investigation, suggesting that the last 40% were exposed to *Mtb* very recently and did not have time to mount adaptive T-cell immunity. The fact that the identified gene signatures did not differentiate between QFT-GIT positive and negative subjects within either incipient TB cases suggests that (innate) immune responses triggered by recent infection/re-infection masks pre-existing adaptive immunity.

Regrettably, our identified signatures cannot be evaluated in other publicly available omics data sets due to partially missing genes (i.e., missing due to differences in sequencing depth covered). In the 11-gene signature, 4 genes were protein-coding (*ABLIM2, HLADRB3, METRNL*, and *UACA*), and 4 genes were long non-coding RNAs (lncRNA; *C20orf197, CTC-543D15.3, RAB11B-AS1*, and *RP4-614C10.2*), and 3 genes were pseudogenes (*CTD-2503O16.3, RNA5SP345*, and *RSU1P1*). Gene description and summary of the 11 identified genes are presented in **Supplementary Table 5**. In the analysis described here, univariate feature selection by t-test identified 145 genes that were of interest. Next, we used pathway analysis to explore the potential function of the 145 identified genes; the gene mapping assessment was linked to the immune system, cytokine signaling, and adaptive immune system. Finally, we also report the continued capacity of the 11-gene signature to separate household contacts categorized as subclinical TB from incipient TB at the 1-year.

The Zak16 (18) signature was adapted to a quantitative RT-PCR, for which the ability to predict TB progression was validated in an independent longitudinal cohort of TB household contacts. Following this, the signature was reduced to 11 genes (RISK11), allowing testing at a 96-well PCR format with equivalent predictor capacity to diagnose co-prevalent TB in household contacts at the time of contact investigation (27). Notably, the capacity of RISK11 to discriminate asymptomatic prevalent TB cases from asymptomatic controls was modest and did not meet the minimal criteria for a triage test. In the study population, where the TB incidence was high and surpassed one case per 100 person-years, the RISK11 signature was able to predict risk for disease progression, but the optimal prognostic performance was limited to a 6-month horizon (27). The 11-gene signature identified in the present study in a population with lower TB incidence could also be useful for predicting TB progression, but further evaluation is required.

Recently, a 5-protein host signature in peripheral blood (C9, IGFBP-2, CD79A, MXRA-7 and NrCAM), called the TB Risk Model 5 (TRM5), predicted disease in South African adolescents within 6 months of TB diagnosis with an AUC 0.96 [95% CI, 0.93–0.99] and within 6–12 months with an AUC 0.76 [95% CI, 0.65–0.87] (41). We also assessed protein host signatures in unstimulated peripheral blood (QFT-GIT Nil-ag supernatants) for the purpose of identifying subclinical TB, representative of household contacts that progressed to TB during the 1-year follow-up. At the time of contact investigation (baseline), the identified 8-protein signature discriminated subclinical TB from incipient TB (household contacts that successfully contained *Mtb* during the 1-year follow-up). Interestingly, the 8-protein signature also discriminated subclinical TB from active TB (Index patients) with a high AUC of 0.93. The eight proteins constituting the signature are reported with diagnostic potential for active TB in previous studies (20, 42–45).

We believe that the combined tools ensure the robustness of our findings for data analysis comprising the well-established machine learning approach – SVM and the Lasso regression model. Both methods use leave-one-out cross-validation for signature prediction. In addition, SVM works relatively well when there is a clear margin of separation between classes, and the latter enhances the prediction accuracy and interpretability of the result.

Transcriptomic signature assessment should also include individuals with previous TB disease, HIV infection, diabetes mellitus, and malnutrition and children and individuals from broader geographical distributions to investigate how these variables affect biomarker performance (17). Any biomarker or biosignature has substantial implementation challenges in terms of feasibility and cost. Further, implementing a mass screen-and-treat TB prevention strategy will necessitate new technologies that can process samples quickly and at high throughput at the point of treatment. Disappointingly, a recent open-label randomized controlled trial did not find reduced TB incidence in adults assigned to TB preventive treatment based on the RISK11 transcriptional signature (27). The devastating effect of the covid-19 pandemic on TB control could, at least in part, be battled by early case detection and targeted preventative and therapeutic treatment where appropriate (2, 3), underlying the urgency of further research within this field. Finally, we cannot rule out that TB re-infection may have occurred during the study follow-up period, leading to a misclassification bias.

The sample size used for the biomarker analysis in this study is admittedly modest, we nevertheless considered it worthwhile to explore the data towards biomarker signature prediction, given the quality of the diagnostic criteria, the stringent diagnostic analysis used for this cohort and the paucity of pre-existing comparable data. To the best of our knowledge, we have not succeeded in identifying other prospective cohort studies that allow for a similar identification of incipient TB cases, a state that is indeed challenging to discriminate from subclinical TB without the proof that longitudinal follow-up provides on whether TB develops or not. Further, a potential misclassification bias is minimized by inclusion of follow-up period and by evaluating the signatures at baseline and follow-up (at 6 or 12 months). Finally, the present nested case-control study utilized samples from patients that are stringently classified using currently available definitions to identify signature(s) that differentiate subclinical from incipient TB. This is a complementary approach to that provided by Gupta RK et al. (29), who performed a systematic meta-analysis using a larger sample size by pooling data from 4 cohorts of adolescent and adult samples and identified eight concise signatures that differentiated incipient TB from healthy controls. Our Incipient TB cases, in keeping with the case definition by Kik SV et al. (15), naturally control the disease with time, whereas in contrast Gupta RK et al., use the notation Incipient TB for subjects who progress to TB. Transcriptomic data from the current study would advance the field of TB biomarker research by inclusion in similar systematic reviews and meta-analyses in the near future.

We acknowledge that the study has limitations; i) the sample size was determined by the availability of samples, which for reasons of logistical challenges could not be obtained from all subjects; therefore, obtaining a larger sample size for the analysis was not possible. ii) We acknowledge that the limited sample size may have affected the level of discrimination reported and imposed a risk of overfitting in this study. However, the identified signature was evaluated in other categories in the TB disease spectrum and could consistently differentiate cases and controls. These limitations highlight the need for more and larger biomarker studies in diverse populations. Finally, there are inherent limitations to using definitive categorical definitions, i.e., for incipient and subclinical TB which are part of the continuous process that the TB disease spectrum represents. Nevertheless, recognizing incipient and subclinical TB as fluctuating or progressive states between latent and active TB along the clinical disease spectrum provides opportunities to develop tools for these stages, which are study attempts to undertake.

In conclusion, to our knowledge, we are the first to explore the genome-wide transcriptional profiling of incipient and subclinical TB. We successfully identified a discriminatory 11- gene signature with high accuracy at baseline contact investigation and on follow-up a year later. Furthermore, we report on the capacity of the very same 11-gene signature to discriminate between subclinical TB vs. active TB and subclinical vs. household controls. Finally, the potential to discriminate across all four distinct stages of the TB disease spectrum is confirmed by PCA analysis. These results point towards a potential to improve the classification of early TB disease states to enable tailored treatment to reduce TB morbidity and transmission by providing care early in the disease trajectory, which would benefit individuals by preventing extensive lung damage and the risk of post-TB sequelae.

## Data availability statement

The datasets presented in this study can be found in online repositories. The names of the repository/repositories and accession number(s) can be found below: https://www.ncbi.nlm.nih.gov/genbank/, GSE193777.

## Ethics statement

Ethical approval for this study was obtained from the Institutional Ethical Review Board (IERB) of St. John’s Medical College, Bangalore (IERB/1/527/08 date 15.07.2008). The material transfer agreement between St. John’s Medical College, Bangalore and the University of Bergen, Norway, was obtained from the Department of Biotechnology, Government of India (No. BT/Med.II/Adv (SS)/Misc./02/2012). In addition, ethical approval was obtained from the Regional Committee for Medical and Health Research Ethics, Western-Norway (REK Vest; Ref no: 2018/1614 D). Written informed consent to participate in this study was provided by the participants’ legal guardian/next of kin.

## Author contributions

DS, SJ, AS, VS, CR, TD, and HG conceptualized and designed the biomarker study. MV coordinated patient recruitment and follow-up. DS and SJ wrote the manuscript with contributions from TD, CR, and HG. DS performed all laboratory experiments (expect RNA-sequencing). AS performed RNA-seq data processing and taking care of the submission of RNA-seq data in the GEO bank. DS performed data analysis and generated Tables and Figures. CR supervised the statistical analysis, wrote the section on statistical analysis, and reviewed the manuscript. HG had primary responsibility for the final content of the manuscript. All authors have contributed to and approved the final manuscript.

## Glossary

**Table:**

*ABLIM2*	Actin Binding LIM Protein Family Member 2
AFB	Acid-fast bacilli
ANOVA	Analysis of variance
AUC	Area under curve
BCG	Bacillus Calmette-Guerin
b-FGF	basic Fibroblast Growth Factor
C9	Complement component 9
*C20orf197*	Chromosome 20 open reading frame 197
CCL	Chemokine C-C motif ligand
CD79A	Cluster of differentiation 79A
cDNA	Complementary deoxyribonucleic acid
*CTC-543D15.3*	Homo sapiens chromosome 19 clone CTC-543D15
*CTD-2503O16.3*	Zinc finger protein processed pseudogene
CXCL	Chemokine C-X-C motif ligand
CXR	Chest X-ray
DEseq	Differential expression analysis for sequence count data
GC6	Grand challenge project no.6 consortium
G-CSF	Granulocyte-colony stimulatory factor
GM, CSF	Granulocyte macrophage-colony stimulatory factor
GO	Gene ontology
HIV	Human immunodeficiency virus;
*HLADRB3*	HLA class II histocompatibility antigen DR beta 3 chain
IFNγ	Interferon gamma
IGFBP-2	Insulin-like growth factor binding protein 2
IGRA	Interferon gamma release assay
IL1RA	Interleukin-1 receptor antagonist
IL	Interleukin
IL12p70	Interleukin 12 subunit p70
IP	Interferon gamma-induced protein 10
KEGG	Kyoto Encyclopedia of genes and genomes
Lasso	Least absolute shrinkage and selection operator;
*METRNL*	Meteorin Like, Glial Cell Differentiation Regulator
mRNA	messenger ribo nucleic acid
*Mtb*	*M. tuberculosis*
MCP	Monocyte chemoattractant protein
MIP	macrophage inflammatory protein
MXRA-7	Matrix Remodeling associated 7
NGS	Next generation sequencing
NrCAM	Neuronal Cell adhesion molecule
PCA	Principal component analysis
PDGF-BB	Platelet-derived growth factor BB protein
PTB	Pulmonary tuberculosis
QFT-GIT	QuantiFERON TB Gold In-tube
RNA	Ribo nucleic acid
*RAB11B-AS1*	RAB11B Antisense RNA 1
RANTES	Regulated on activation Normal T cell expressed and secreted
RIN	RNA intensity number
ROC	Receiver operating characteristic
*RP4-614C10.2*	Long intergenic non-protein coding RNA 2802 is an RNA Gene
*RNA5SP345*	RNA 5S Ribosomal Pseudogene 345
RNTCP	Revised National Tuberculosis Control Program
RT-PCR	Reverse transcription polymerase chain reaction
*RSU1P1*	Ras Suppressor Protein 1 Pseudogene 1
SVM	Support vector machine
TNF	Tumor necrosis factor
TPP	Target product profile
TRM5	TB Risk Model 5
TST	Tuberculin skin test
*UACA*	Uveal Autoantigen with Coiled-Coil Domains and Ankyrin Repeats
VEGF	Vascular endothelial growth factor
WebGestalt	WEB-based GEne SeT AnaLysis Toolkit
WHO	World Health Organization
