# Supplementary material for: Host blood-based biosignatures for subclinical TB and incipient TB: A prospective study of adult TB household contacts in Southern India
Source: Front Immunol. 2023 Jan 11;13:1051963. doi: 10.3389/fimmu.2022.1051963 (PMC9876034; doi:10.3389/fimmu.2022.1051963)
Supplement: Supplementary file 1 [file Presentation_1.pptx]

## Slide 1
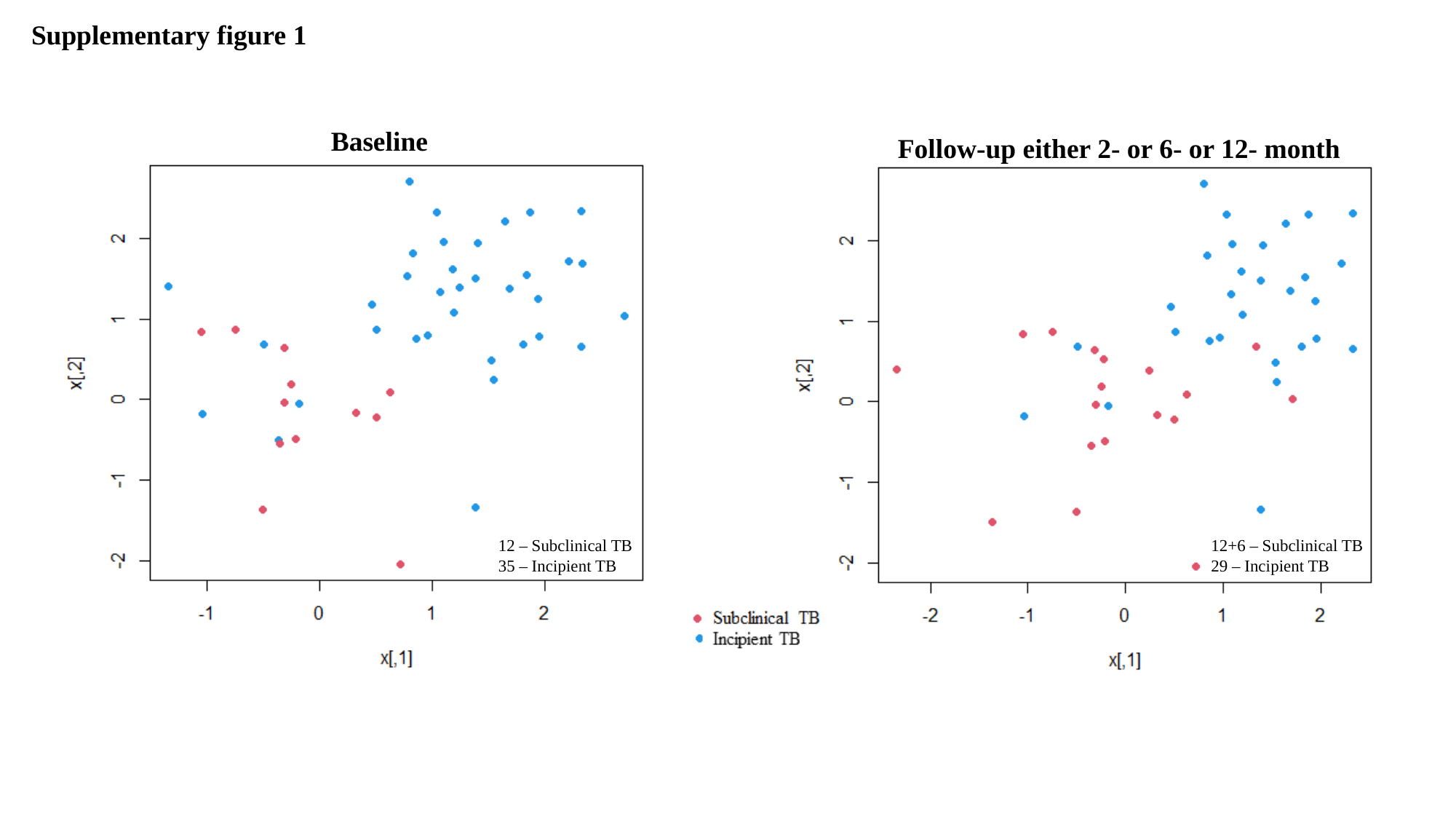

Supplementary figure 1
Baseline
Follow-up either 2- or 6- or 12- month
12 – Subclinical TB
35 – Incipient TB
12+6 – Subclinical TB
29 – Incipient TB

## Slide 2
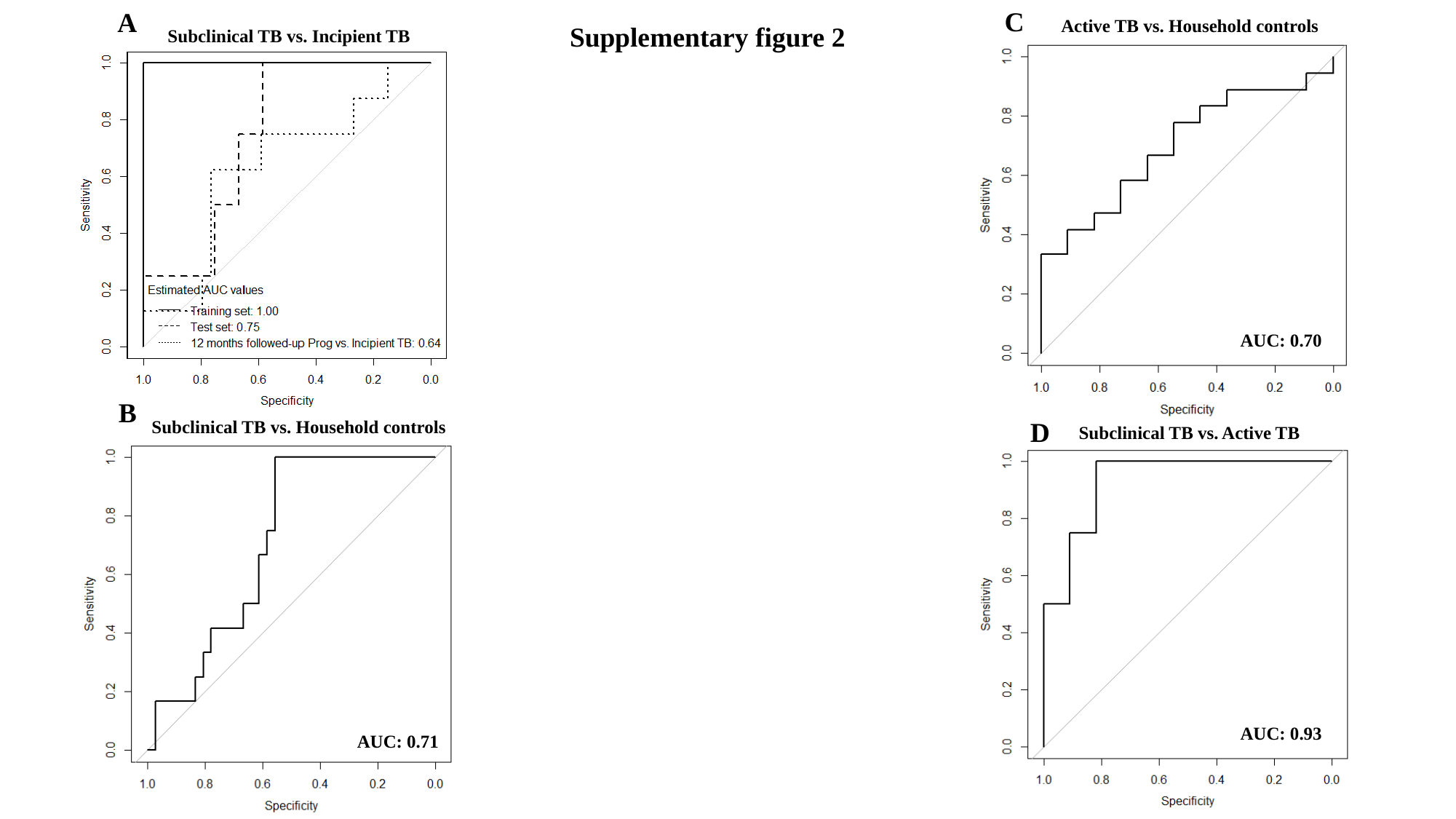

A
Subclinical TB vs. Incipient TB
C
Active TB vs. Household controls
Supplementary figure 2
AUC: 0.70
B
Subclinical TB vs. Household controls
AUC: 0.71
D
Subclinical TB vs. Active TB
AUC: 0.93

## Slide 3
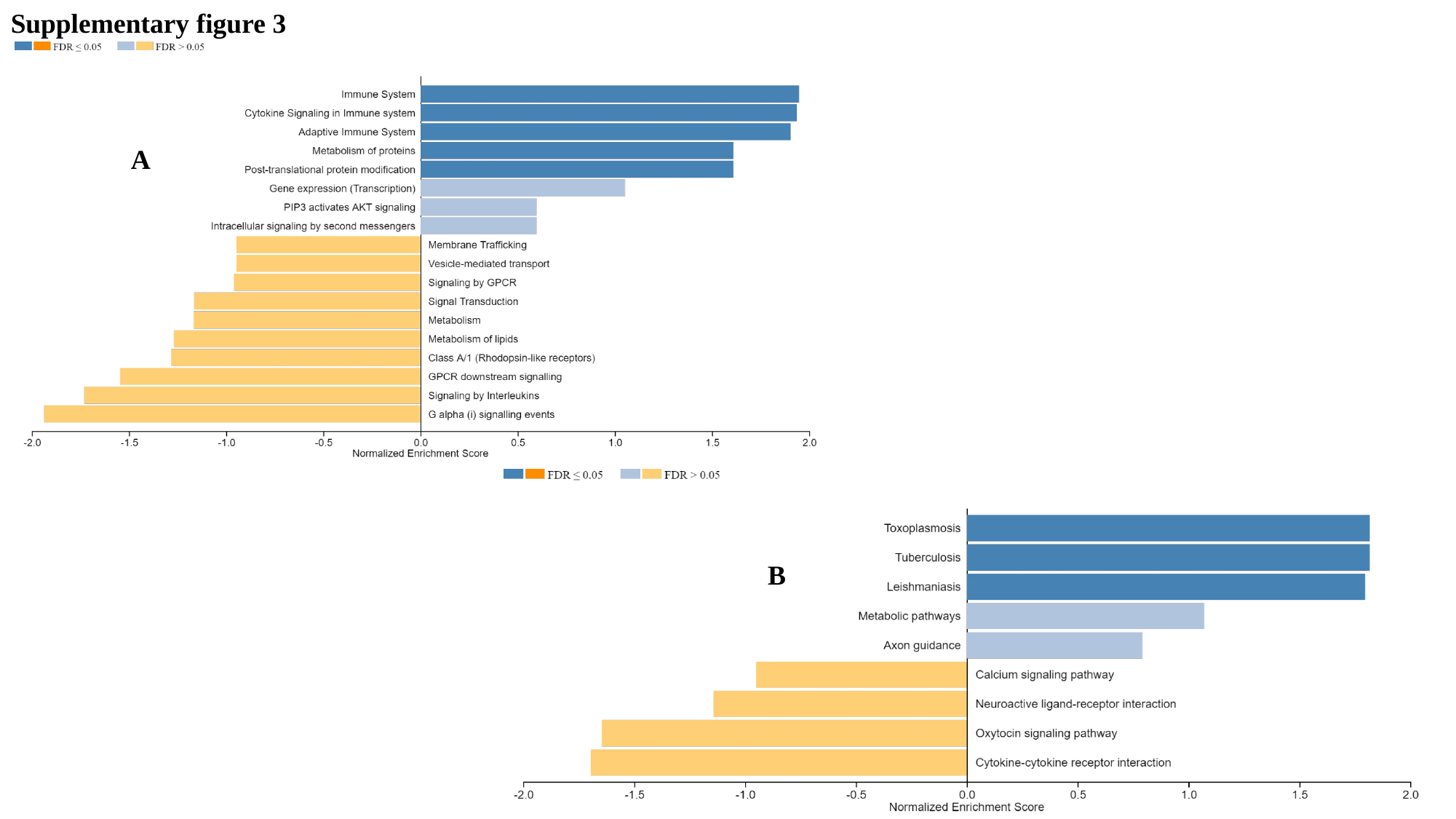

Supplementary figure 3
A
B
